# Electrochemotherapy of tumours

**DOI:** 10.3747/co.v16i2.368

**Published:** 2009-03

**Authors:** G. Sersa, M. Cemazar, M. Snoj

**Keywords:** Electrochemotherapy, melanoma, electroporation

Several novel tumour-targeting and drug-delivery approaches in cancer treatment are currently undergoing intensive investigation, among them physical approaches such as tissue electroporation. Since the early 1980s, electroporation has proved to be effective in facilitating the transport of various molecules across the plasma membrane. By using short, intense electric pulses, the plasma membrane becomes permeable to molecules such as bleomycin and cisplatin that are otherwise deprived of membrane transport mechanisms. The most appropriate electric pulses are those of a duration and amplitude suitable to preserving the viability of cells, allowing for resealing of the cell membrane a few minutes after the application of the pulses^1^.

Electroporation-based cancer treatment approaches are currently undergoing intensive investigation in the field of drug delivery and gene therapy. The first biomedical application of this method of treating cancer came in the form of electrochemotherapy, which, since its beginnings in the late 1980s, has evolved into a clinically verified treatment approach for cutaneous and subcutaneous tumour nodules 2.

## DEFINITION AND MECHANISM OF ELECTROCHEMOTHERAPY

Electrochemotherapy is defined as a local treatment that, through the application of cell-membrane-permeabilizing electric pulses, potentiates at the application site the high intrinsic cytotoxicity of non-permeant or poorly permeant anticancer drugs. Among several drugs that have been tested to date, only two drugs have been identified as candidates for electrochemotherapy of cancer patients: bleomycin and cisplatin 3.

The transport of bleomycin across the nonpermeabilized plasma membrane is achieved by carrier proteins that internalize it by the endocytotic pathway, but that transport is limited by the low number of carrier proteins. Using electroporation to increase membrane permeability provides bleomycin with direct access to cytosol and transport to dna. The cytotoxicity of bleomycin is thereby increased by a multiple of several thousand 3.

Cisplatin transport through the nonpermeabilized plasma membrane is also normally hampered. Only 50% of cisplatin is transported through the plasma membrane by passive diffusion; the rest is transported by carrier molecules. Electroporation of the plasma membrane enables increased flux and accumulation of the drug in cells, which results in an increase in cisplatin cytotoxicity by a multiple of up to 80 4.

The principal mechanism of electrochemotherapy is the electroporation of cells in tumours, a process that increases drug effectiveness by enabling the drug to reach the intracellular targets. Other mechanisms of electrochemotherapy—for example, prolonged drug entrapment in tumours because of a transient induced but reversible reduction of tumour blood flow, and a vascular disruption effect are also involved, contributing to the overall effectiveness of the treatment 3,5,6.

## CLINICAL USES

The first clinical study of electrochemotherapy, involving head-and-neck tumour nodules, was published in 1991 2; several others followed thereafter 7. These clinical studies demonstrated the antitumour effectiveness of electrochemotherapy with either bleomycin or cisplatin, given intravenously or intratumorally. A response was demonstrated in single and multiple cutaneous or subcutaneous melanoma nodules, in breast and head-and-neck cancer nodules, and in Kaposi sarcoma, hypernephroma, chondrosarcoma, and basal cell carcinoma 7. Recently, a prospective multi-institutional study was conducted by a consortium of four cancer centers gathered in the European ESOPE (European Standard Operating Procedures of Electrochemotherapy) project. The results confirmed the previous reports that electrochemotherapy is effective in the treatment of cutaneous and subcutaneous tumour nodules with an 85% objective response rate (74% complete response rate), regardless of tumour histology and of drug (bleomycin, cisplatin) or route of administration used 8. In addition, standard operating procedures for electrochemotherapy were published 9.

Currently, electrochemotherapy is being used in approximately 40 cancer centres in Europe and the United States. Several hundred patients were successfully treated in 2008. Current clinical use of electrochemotherapy is focused on palliation of progressive disease; however, it can also be used as cytoreductive treatment before surgical resection in an organ-sparing attempt. It has been used in such a setting before a sphincter-sparing resection of anal melanoma and in digital chondrosarcoma, rescuing the finger from amputation. Because of its vascular disruption effect, electrochemotherapy is also effective in the treatment of bleeding metastases 5. Furthermore, it could be used to treat basal cell carcinoma of the face with curative intent. Its beneficial antitumour effects have been proved, giving better cosmetic results than excisional surgery does 10.

## CURRENT DEVELOPMENTS

Current developments and future medical applications of tissue electroporation are numerous. Because electrochemotherapy with bleomycin or cisplatin has been shown to act synergistically with radiotherapy in preclinical studies, use of the technique for the radiosensitization of cutaneous tumours can be foreseen, predominantly in the palliative treatment of progressive disease.

The use of electroporation technology in gene therapy—moving various kinds of nucleic acids (small interfering rna, plasmid dna, oligonucleotides) into cells—is called “gene electrotransfer.” This approach needs further development to achieve better results, but its clinical use can be foreseen in the near future 11. The first phase i and ii clinical studies are ongoing 12.

Further development is focused on the use of endoluminal electrodes to treat internal tumours, a technological development that could be used for either electrochemotherapy or gene electrotransfer 5,7.
